# Analysis of Joint Power and Work During Gait in Children With and Without Cerebral Palsy

**DOI:** 10.1007/s43465-022-00691-8

**Published:** 2022-07-14

**Authors:** Priyam Hazra, Sheila Gibbs, Graham Arnold, Sadiq Nasir, Weijie Wang

**Affiliations:** Institute of Motion Analysis and Research (IMAR), Department of Orthopaedic and Trauma Surgery, Ninewells Hospital and Medical School, University of Dundee, Dundee, DD1 9SY UK

**Keywords:** Cerebral palsy, Healthy children, Joint power, Work, Energy

## Abstract

**Purpose:**

To compare joint work in the lower limb joints during different sub-phases of the gait cycle between Cerebral Palsy (CP) and healthy children.

**Methods:**

Eighteen CP and 20 healthy children’s gait data were collected. The CP group included orthoses, intra-muscular injection of botulinum toxin and surgery groups. A motion capture system was used to collect gait data. Joint work was calculated as positive and negative components in six subphases during gait and normalised by speed when comparing the groups.

**Results:**

The CP group had a slower walking speed, smaller stride length and longer stance phase than the healthy group. Hip max positive work was 0.12 ± 0.02 Jkg^−1^/ms^−1^ for the CP group in pre-mid-stance but 0.07 ± 0.01 Jkg^−1^/ms^−1^ for the healthy group during the terminal phase. In terminal stance, ankle positive work was significantly lower in the CP group (0.12 ± 0.01) than in the healthy group (0.18 ± 0.01). The knee showed a similar distribution of positive work in the stance phase for the two groups. In the ankle and hip, the CP group had energy generation mainly in midstance while the healthy group was mainly in terminal stance. In the ankle, the CP group had larger energy absorption in mid-stance than the healthy children group, while the CP group showed lower energy generation in the terminal stance phase than seen in the healthy group.

**Conclusion:**

The qualitative and quantitative analysis of joint work provides useful information for clinicians in the treatment and rehabilitation of CP patients.

## Introduction

Cerebral palsy (CP) is a common disorder of movement and posture in children. It affects 2–2.5 in every 1000 live births according to Reddihough and Collins (2003) [1]. It has been found that children with CP who exhibit a crouch gait have increased tone, that is, spasticity, in the hip adductors, flexors, hamstring muscle tightness and stretched calf muscles. They show weakness in adequate force generation, loss of selective control and slow movements (negative features) according to Graham and Selber (2003) [2]. Thus, they have multi-level involvements (Brunner and Rutz 2013) [3].

Three-dimensional motion analysis, foot pressure measurement, surface Electromyography (sEMG) and oxygen consumption during walking have been used to study different kinematic and kinetic parameters of body segments during gait (Armand et al. 2016; Baker, 2013; Gage, 1993; Hirschmann et al. 2016; Muhammad et al. 2015) [4–8]. Gait analysis helps us understand the effect of one segment upon another body segment or joint before and after treatment in CP (Makaram et al. 2018; Sung et al. 2018; Pilloni et al. 2018; Sees et al. 2018; Wang et al. 2018) [9–13]. However, it is not clear how joint power and work are used in different phases of the gait cycle in a child with CP. Ishihara and Higuchi (2014) studied the peak hip flexor power generation in spastic hemiplegic and healthy children, however, the number of participants was small and did not include other types of CP [14]. Dohin and Salem (2015) studied the hip and ankle power generation 1-year post-SEMLS surgery, however, they did not compare their results with healthy children [15]. Booth et al. (2018) studied step length, knee extension and ankle power in children with CP without comparison to a healthy group [16]. So far, there is little research conducted on joint power and work during gait cycle phases (Schless et al. 2019; Kalita et al. 2020; Waterval et al. 2018) [17–19]. The purpose of this study was two-fold: firstly, to calculate joint power and work in the hip, knee and ankle joints in sub-phases during gait; and secondly, to compare these results between children with and without CP in terms of timings and quantities.

## Methods

This was a retrospective study comprised of three-dimensional gait analysis data of 18 CP and 20 healthy children. After gaining Caldicott approval, the gait data were extracted from the existing database of Motion and Gait Analysis lab in the University Department of Orthopaedic and Trauma Surgery, Ninewells Hospital and Medical School, University of Dundee. The lab holds a general ethical approval by the local hospital and institutional research ethics committee and conformed to the Helsinki Declaration. All participants signed written consent forms when they attended to data collection.

The inclusion criteria were that (1) subjects were able to walk without a walking aid, (2) their marker and force plate data were complete, (3) their clinical treatments would be any. The CP patients included various types, e.g., ankle/foot orthoses (AFO, *n* = 15 limbs), surgery (*n* = 9 limbs), botulinum toxin (Botox, *n* = 17 limbs), and thus some participants shared more than one treatment. The CP gait data was collected while participants were barefoot and without using a walking aid. The control group comprised 20 healthy children.

All participants had undergone gait analysis using a three-dimensional motion measurement system (Vicon^®^ Nexus 2.8) for data collection and processing (Oxford Metrics Limited, Oxford, UK). The infra-red light reflected from 14 mm retro-reflective markers taped on the participants’ body was captured by 14 infra-red cameras and was transferred in an electronic format to the computer installed with Vicon^®^ Nexus 2.8 software. The two-dimensional images captured by cameras were converted into three-dimensional stick figures in the software frame by using participants’ anthropometric measurements, labelling markers, defining the gait cycle and running the dynamic Plug-in-Gait model provided by Vicon^®^. A minimum of 10 trials were conducted in each session, out of which three good trials were selected for analysis. The gait data were obtained from a clinical gait laboratory which has routinely been used to collect CP gait for more than 20 years.

### Power, Work and Energy Calculations

Firstly, the whole gait cycle was divided into stance and swing phases. Then, the stance phase was divided into four phases, i.e., loading phase (0–1/6), pre-mid-stance (1/6–3/6), post-mid-stance (3/6–5/6) and terminal stance (5/6–6/6). The reason for dividing this way is that the stance phase usually makes up 60% of the gait cycle. The swing phase was further divided into three phases i.e., initial-swing, mid-swing and terminal swing or pre-stance, each taking 30% of the swing phase. For each phase, five parameters were calculated, i.e., sum of positive work, sum of negative work, sum of absolute work, and maximum and minimum powers. In terms of physical meaning, work done (Joule/kg) by the joints was indicated to energy (Joule/kg) observed during gait. Therefore, the signs of work indicate that the quantity of work is generated (positive) or absorbed (negative). From the Plug-in-Gait model, joint power (Watt/kg) was calculated using the product of the joint moment and angular velocity, but joint work was not calculated by the Plug-in-Gait. Therefore, an in-house software program was developed to calculate joint work in Joule/kg for different phases of the gait cycle. The equations used to calculate work and power are as below, mainly according to [22, 23].

Firstly, power is equal to the product of the joint moment and angular velocity as (1):

1 $$P = M\,\omega$$

Where *P* is joint power, *M* joint moment and *ω* angular velocity. According to biomechanics, increment of work is equal to the product of joint moment and increment of angles as:

2 $$dW = M{\kern 1pt} d\alpha$$

Where *α* is joint angle and *W* joint work. The Eq. (2) can be written as (3):

3 $$\frac{dW}{{dt}} = M\frac{d\alpha }{{dt}}$$

Where *dt* is the time between two interval frames in gait data. Combining the equations of (1−3), we get the equation for calculating joint work as (4).

4 $$W = \sum Pdt$$

**Fig. 1a:**
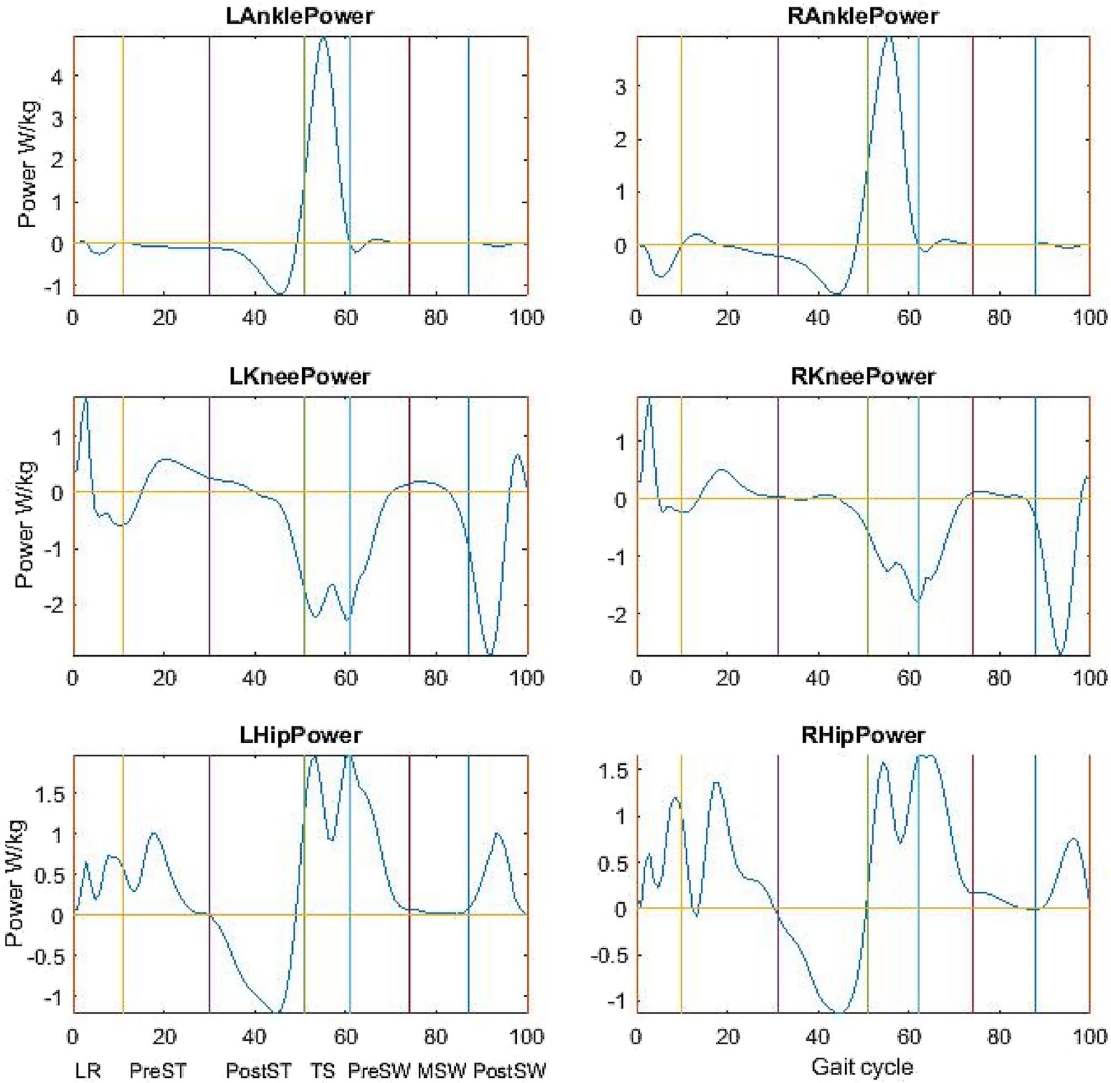

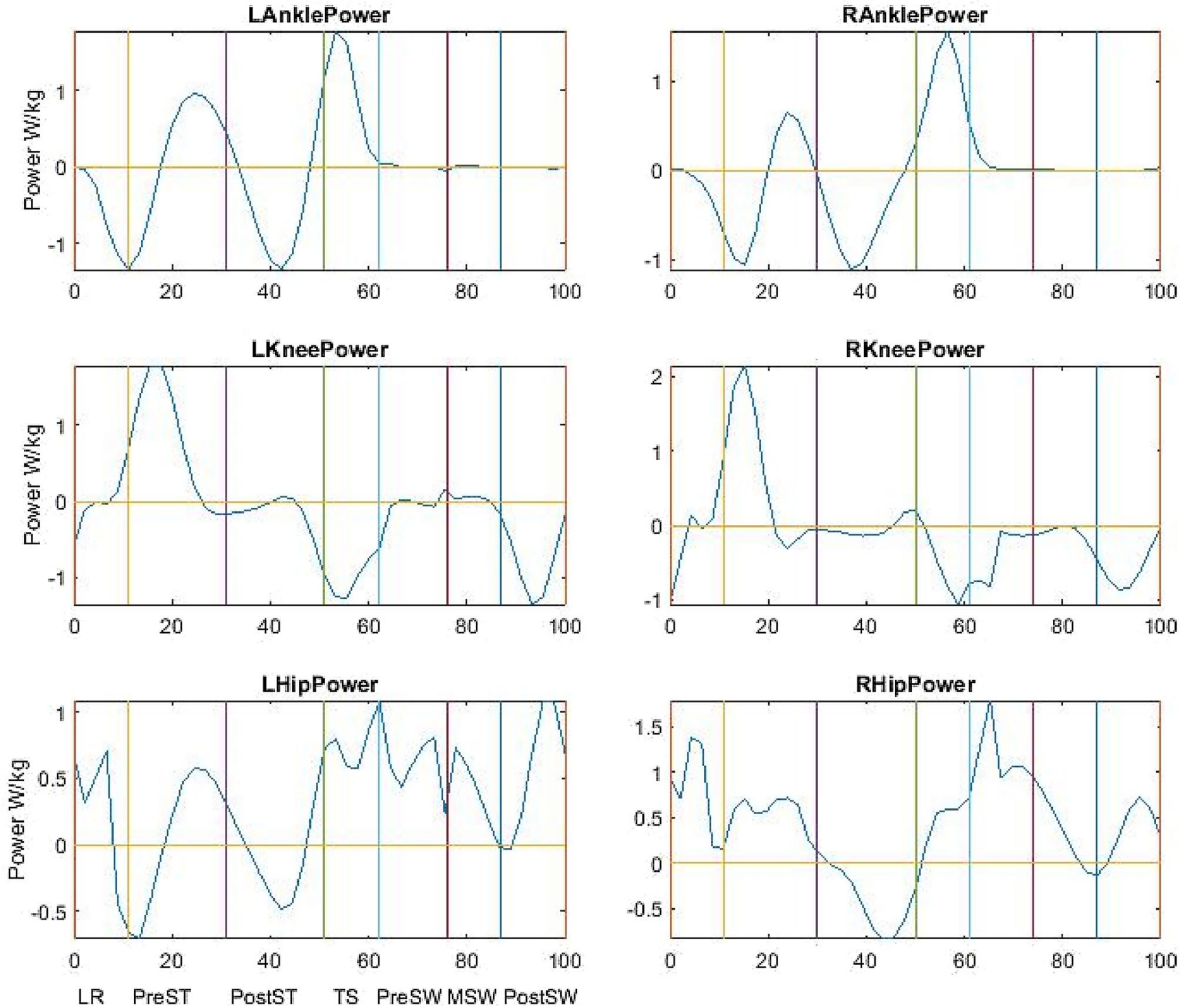
a A set of typical power curves from a healthy participant. Note: The gait cycle starts from heel strike and finishes at next heel strike; the reference lines divide a gait cycle into seven phases, i.e. loading response (LR), pre-stance (PreST), post-stance (PostST), terminal stance (TS) and three swing phases, and joint power and work were calculated for each phase with both positive and negative components. b A set of typical power curves from a patient with cerebral palsy. Note: The gait cycle starts from heel strike and finishes at next heel strike; the reference lines divide a gait cycle into seven phases, i.e. loading response (LR), pre-stance (PreST), post-stance (PostST), terminal stance (TS) and three swing phases, and joint power and work were calculated for each phase with both positive and negative components

Thus, we can calculate joint work from joint power. The sign of Σ is used to replace the integral for specific time periods, e.g., positive, negative or all values in a phase, depending on the parameters to be calculated. The calculation was performed by the in-house program made in MATLAB^®^ and power graphs were acquired (see Fig. 1). As walking speeds were different from individuals, all joint work and power were normalised by dividing respective walking speeds. All power and work were also normalised by body mass. The normalised joint work and power were analysed and compared.

### Statistical Method for Analysis

The Statistical Package for Social Sciences (SPSS, IBM, USA) version 25 software was used to compare spatial–temporal parameters, joint power, positive work, negative work, and the absolute sum of work between two groups in terms of mean values and standard deviation. The statistical test of difference was the general linear model for multi-variate which is similar to an independent *t*-test but suitable for multi-repeated trials. The level of significant difference was set as *p* < 0.05. Data for right and left side limbs were considered per participant.

## Results

Twenty children were selected for participation, 13 males and 7 females, 1 below 7 years of age and the average age of healthy children was 11 years with a range of 6–15 years at the time trials were collected. The CP group consisted of 11 male and 7 female children. The average age of patients was 9 years old with a range of 4–22 years old at the time of trials. The children were diagnosed as hemiplegic (1 right, 2 left, total 3), diplegic (asymmetric 1, spastic 10, total 11), quadriplegic (2), and ataxic (1). Diplegia (*n* = 11) was the most common diagnosis. The ataxic type of CP was the least diagnosed type (*n* = 1). Most children were treated with Botox (41%) in the present study and surgical treatment was the least (22%). However, treatment with surgery may also include treatment with AFO or Botox or a combination of both. There were 15 children treated with AFO, 10 males and 5 females. Ten of them wore AFO bilaterally (67%) and the remaining 5 unilaterally (3 left, 2 right). There were 17 children who were treated with Botox injection, 12 male (67%) and 5 female (33%). There were 14 bilateral (74%) and 5 unilateral treatment groups. The muscles injected were hamstrings (right 9, left 10), gastrocnemius (right 9, left 10), rectus femoris (right 8, left 8), adductus magnus (left 1), tendon Achilles (right 1, left 1), tibialis posterior (*n* = 1) and multi-level injection (*n* = 1). Hamstrings muscle was the target injection site in the highest number of children (*n* = 12) while the tibialis posterior injection was given to only one child. Surgical history could be obtained from 9 selected children’s data files. Among them, males were 4 and females were 5. There were 8 bilateral (62%) and 5 unilateral (38%) groups. The surgical procedures carried out were either osteotomies or soft tissue surgeries. Proximal femoral osteotomy was the mostly done bony operation (*n* = 7 limbs) while gastrocnemius recession (*n* = 6 limbs) and hamstring lengthening (*n* = 6 limbs) both equally qualified for the mostly performed soft tissue operation as in Table 1. Some patients had mixed symptoms. It should be noted that all patients were able to walk independently without the use of walking aids, and their gait data were collected when they walked independently.

**Table 1:** Demographic details of the group of CP children and healthy group

	Children without CP	Children with CP	Note
Sample size	20	18	
Treatment	n/a	AFO/Botox/Surgery	
Age median (range)	11 (6–15)	9 (4–22)	
Gender	13 M 7F	11 M 7F	
Clinical type	n/a	Hemiplegic = 3 diplegic = 11 quadriplegic = 3 ataxic = 1	
Body mass (m) mean (range)	38.7 (20–59)	41.7 (17–82)	*p* = 0.556
Height (m) mean (range)	1.46 (1.22–1.77)	1.46 (1.07–1.79)	*p* = 0.993

*Clinical information from NHS files. Original data was collected from 2007 to 2012. All children with CP were able to walk independently.

### Spatial–temporal Parameters

Statistical difference between CP and healthy groups was noted in terms of walking speed, foot off, stride length, and step length. Usually, CP children have slower walking speed, longer stance, shorter stride length, and shorter step length than healthy children, but similar cadence as shown in Table 2. It was also found that after any form of treatment, the children with CP could not achieve a near healthy state.

**Table 2:** Differences in spatial–temporal parameters between CP and healthy children

Variable	Group	Mean	Std. Deviation	*p*
Walking Speed (m/s)	CP	1.04	0.26	< 0.001
	Healthy	1.30	0.18	
Cadence (step/min)	CP	122.30	16.66	0.9630
	Healthy	122.49	22.71	
Stance or foot off (%)	CP	61.36	4.52	< 0.001
	Healthy	58.49	2.28	
Stride length (m)	CP	1.02	0.20	< 0.001
	Healthy	1.30	0.23	
Step length (m)	CP	0.46	0.20	0.0035
	Healthy	0.59	0.25	

The number of trials: CP *n* = 59, healthy *n* = 41; it should be noted that ‘*n*’ is the number of gait trials rather than the number of subjects.

### Ankle Power and Work Done

The results regarding the ankle are shown in Table 3. A statistically significant difference was observed between the CP and healthy groups. As a whole in the stance phase, the sum of absolute work in CP is 0.36 (Jkg^−1^/ms^−1^), similar to the healthy group of 0.34 (Jkg^−1^/ms^−1^), but the CP had higher values in the first three sub-phases of stance while the healthy group displayed higher values in the terminal stance only. This work distribution showed a different way of spending energy between both groups of children.

**Table 3:** Comparison of ankle power and work done in different phases of the gait cycle between CP (*n* = 61) and healthy children (*n* = 42)

Ankle joint	Loading response (*p* value)	Pre-mid stance (p)	Post-mid stance (p)	Terminal stance (p)	Initial swing(p)	Mid-swing(p)	Terminal swing(p)
Maximum power	CP = 0.21 ± 0.085 NC = 0.30 ± 0.102 (0.481)	CP = 0.31 ± 0.053 NC = 0.17 ± 0.063 (0.112)	CP = 1.01 ± 0.114 NC = 1.09 ± 0.136 (0.627)	CP = 1.72 ± 0.147 NC = 2.77 ± 0.176 (*p* < 0.0001**)	CP = 0.22 ± 0.045 NC = 0.37 ± 0.055 (0.038*)	CP = 0.03 ± 0.011 NC = 0.03 ± 0.013 (0.927)	CP = 0.04 ± 0.030 NC = 0.08 ± 0.036 (0.371)
Minimum power	CP = − 0.68 ± 0.094 NC = − 0.52 ± 0.113 (0.265)	CP = − 0.79 ± 0.099 NC = − 0.44 ± 0.119 (0.025*)	CP = − 0.63 ± 0.059 NC = − 0.35 ± 0.071 (0.003*)	CP = 0.06 ± 0.043 NC = 0.19 ± 0.052 (0.059*)	CP = − 0.07 ± 0.024 NC = − 0.15 ± 0.029 (0.057)	CP = − 0.02 ± 0.009 NC = − 0.01 ± 0.011 (0.355)	CP = − 0.02 ± 0.078 NC = − 0.28 ± 0.093 (0.061)
Sum positive work	CP = 0.01 ± 0.004 NC = 0.01 ± 0.004 (0.814)	CP = 0.03 ± 0.004 NC = 0.01 ± 0.005 (0.025*)	CP = 0.05 ± 0.006 NC = 0.04 ± 0.007 (0.475)	CP = 0.12 ± 0.010 NC = 0.18 ± 0.012 (*p* < 0.0001**)	CP = 0.01 ± 0.001 NC = 0.01 ± 0.001 (0.028*)	CP = 0.001 ± 0.001 NC = 0.002 ± 0.001 (0.736)	CP = 0.001 ± 0.001 NC = 0.003 ± 0.001 (0.367)
Sum negative work	CP = − 0.04 ± 0.005 NC = − 0.02 ± 0.006 (0.028*)	CP = − 0.06 ± 0.007 NC = − 0.04 ± 0.008 (0.090)	CP = − 0.06 ± 0.006 NC = − 0.04 ± 0.007 (0.006*)	CP = − 0.001 ± 0.001 NC = 0.001 ± 0.001 (0.826)	CP = − 0.004 ± 0.002 NC = − 0.003 ± 0.002 (0.852)	CP = − 0.001 ± 0.000 NC = − 0.000 ± 0.000 (0.419)	CP = − 0.001 ± 0.002 NC = − 0.01 ± 0.002 (0.059)
Absolute sum of work	CP = 0.05 ± 0.006 NC = 0.03 ± 0.007 (0.037*)	CP = 0.08 ± 0.008 NC = 0.05 ± 0.010 (0.009*)	CP = 0.11 ± 0.007 NC = 0.08 ± 0.008 (0.002*)	CP = 0.12 ± 0.010 NC = 0.18 ± 0.012 (*p* < 0.0001**)	CP = 0.01 ± 0.002 NC = 0.01 ± 0.003 (0.466)	CP = 0.002 ± 0.001 NC = 0.002 ± 0.001 (0.962)	CP = 0.002 ± 0.002 NC = 0.01 ± 0.003 (0.103)

*CP* cerebral palsy, *NC* healthy children, *Power* Watt kg^−1^/ms^−1^, *Work* Joule kg^−1^/ms.^−1^

*p < 0.05, **p < 0.0001.

### Knee Power and Work Done

A statistically significant difference was observed between the CP and healthy groups of children as shown in Table 4. The knee had similar positive work distributions in the two groups in the stance phase, but the negative sum of CP was 0.18 (Jkg^−1^/ms^−1^) while the healthy group was − 0.22 (Jkg^−1^/ms^−1^), which indicates that the healthy knee absorbed energy better than the CP group in stance.

**Table 4:** Comparison of knee power and work done in different phases of the gait cycle between CP (*n* = 61) and healthy children (*n* = 42)

Knee joint	Loading response (*p* value)	Pre-mid stance (*p*)	Post-mid stance (*p*)	Terminal stance (*p*)	Initial swing(*p*)	Mid-swing(*p*)	Terminal swing(*p*)
Maximum power	CP = 0.75 ± 0.120 NC = 0.97 ± 0.144 (0.235)	CP = 1.16 ± 0.110 NC = 0.89 ± 0.132 (0.121)	CP = 0.14 ± 0.021 NC = 0.15 ± 0.025 (0.875)	CP = − 0.14 ± 0.046 NC = − 0.48 ± 0.056 (*p* < 0.0001**)	CP = 0.11 ± 0.023 NC = 0.06 ± 0.028 (0.138)	CP = 0.18 ± 0.127 NC = 0.29 ± 0.153 (0.564)	CP = 0.10 ± 0.139 NC = 0.55 ± 0.166 (0.038*)
Minimum power	CP = − 0.74 ± 0.119 NC = − 1.40 ± 0.142 (0.001*)	CP = − 0.57 ± 0.080 NC = − 0.85 ± 0.096 (0.029*)	CP = − 0.62 ± 0.060 NC = 0.67 ± 0.073 (0.614)	CP = − 0.86 ± 0.073 NC = − 1.45 ± 0.087 (*p* < 0.0001**)	CP = − 0.42 ± 0.045 NC = − 0.97 ± 0.054 (*p* < 0.0001**)	CP = − 0.27 ± 0.037 NC = − 0.43 ± 0.044 (0.007*)	CP = − 0.69 ± 0.110 NC = − 1.64 ± 0.132 (*p* < 0.0001*)
Sum positive work	CP = 0.03 ± 0.004 NC = 0.02 ± 0.005 (0.449)	CP = 0.09 ± 0.008 NC = 0.08 ± 0.009 (0.417)	CP = 0.01 ± 0.001 NC = 0.01 ± 0.002 (0.551)	CP = 0.002 ± 0.001 NC = 0.001 ± 0.001 (0.455)	CP = 0.007 ± 0.001 NC = 0.001 ± 0.001(0.006*)	CP = 0.01 ± 0.006 NC = 0.01 ± 0.007 (0.693)	CP = 0.01 ± 0.011 NC = 0.02 ± 0.013 (0.678)
Sum negative work	CP = − 0.04 ± 0.005 NC = − 0.06 ± 0.006 (0.001*)	CP = − 0.03 ± 0.003 NC = − 0.02 ± 0.004 (0.118)	CP = − 0.05 ± 0.005 NC = − 0.03 ± 0.006 (0.004*)	CP = − 0.06 ± 0.006 NC = − 0.11 ± 0.007 (*p* < 0.0001**)	CP = − 0.02 ± 0.002 NC = − 0.06 ± 0.003 (*p* < 0.0001**)	CP = − 0.01 ± 0.002 NC = − 0.01 ± 0.002 (0.760)	CP = − 0.06 ± 0.005 NC = − 0.10 ± 0.006 (*p* < 0.0001**)
Absolute sum of work	CP = 0.06 ± 0.007 NC = 0.09 ± 0.008 (0.022*)	CP = 0.11 ± 0.009 NC = 0.10 ± 0.011 (0.211)	CP = 0.06 ± 0.005 NC = 0.03 ± 0.006 (0.002*)	CP = 0.07 ± 0.006 NC = 0.11 ± 0.007 (*p* < 0.0001**)	CP = 0.03 ± 0.002 NC = 0.06 ± 0.003 (*p* < 0.0001*)	CP = 0.02 ± 0.006 NC = 0.03 ± 0.007 (0.623)	CP = 0.07 ± 0.011 NC = 0.12 ± 0.013 (0.014*)

*CP* cerebral palsy, *NC* healthy children, *Power* Watt kg^−1^/ms^−1^, *Work* Joule kg^−1^/ms^−1^)

*p <0.05, **p <0.0001

### Hip Power and Work Done

A statistically significant difference was observed between the CP and healthy groups of children as shown in Table 5. As a whole, the CP group had the sum of positive work in the stance phase of approximately 0.21 (Jkg^−1^/ms^−1^) while the healthy group was 0.15 (Jkg^−1^/ms^−1^), indicating that the children with CP generate more energy than the healthy children in stance. Alternatively, the CP group had higher positive work in the first half of stance while the healthy group was higher in the second half, indicating that the two groups had different ways to use energy.

**Table 5:** Comparison of hip power and work done in different phases of the gait cycle between CP (*n* = 61) and healthy children (*n* = 42)

Hip joint	Loading response (*p*)	Pre-mid stance (*p*)	Post-mid stance (*p*)	Terminal stance (*p*)	Initial swing(*p*)	Mid-swing(*p*)	Terminal swing(*p*)
Maximum power	CP = 0.62 ± 0.050 NC = 0.48 ± 0.060 (0.070)	CP = 0.97 ± 0.086 NC = 0.32 ± 0.104 (*p* < 0.0001**)	CP = 0.40 ± 0.065 NC = 0.08 ± 0.078 (0.002*)	CP = 0.67 ± 0.055 NC = 0.99 ± 0.066 (*p* < 0.0001**)	CP = 0.80 ± 0.056 NC = 1.20 ± 0.067 (*p* < 0.0001**)	CP = 0.43 ± 0.030 NC = 0.13 ± 0.036 (*p* < 0.0001**)	CP = 0.31 ± 0.046 NC = 0.28 ± 0.055 (0.720)
Minimum power	CP = − 0.27 ± 0.061 NC = − 0.45 ± 0.073 (0.068)	CP = − 0.17 ± 0.065 NC = − 0.49 ± 0.079 (0.002*)	CP = − 0.66 ± 0.050 NC = − 0.82 ± 0.060 (0.046*)	CP = − 0.25 ± 0.056 NC = − 0.12 ± 0.067 (0.139)	CP = 0.05 ± 0.051 NC = − 0.03 ± 0.061 (0.326)	CP = − 0.14 ± 0.091 NC = − 0.13 ± 0.109 (0.909)	CP = − 0.16 ± 0.029 NC = − 0.11 ± 0.035 (0.272)
Sum positive work	CP = 0.03 ± 0.003 NC = 0.02 ± 0.003 (0.001*)	CP = 0.12 ± 0.011 NC = 0.03 ± 0.013 (*p* < 0.0001**)	CP = 0.02 ± 0.003 NC = 0.003 ± 0.003 (*p* < 0.0001**)	CP = 0.04 ± 0.004 NC = 0.07 ± 0.005 (*p* < 0.0001**)	CP = 0.07 ± 0.004 NC = 0.08 ± 0.005 (0.017*)	CP = 0.03 ± 0.003 NC = 0.01 ± 0.004 (*p* < 0.0001**)	CP = 0.02 ± 0.003 NC = 0.02 ± 0.004 (0.654)
Sum negative work	CP = − 0.01 ± 0.003 NC = − 0.01 ± 0.003 (0.885)	CP = − 0.01 ± 0.005 NC = − 0.04 ± 0.006 (0.002*)	CP = − 0.07 ± 0.006 NC = − 0.10 ± 0.007 (*p* < 0.0001**)	CP = − 0.01 ± 0.003 NC = − 0.004 ± 0.003 (0.076)	CP = − 0.01 ± 0.003 NC = − 0.002 ± 0.003 (0.222)	CP = − 0.01 ± 0.007 NC = − 0.01 ± 0.009 (0.536)	CP = − 0.01 ± 0.002 NC = − 0.004 ± 0.002 (0.029*)
Absolute sum of work	CP = 0.05 ± 0.003 NC = 0.03 ± 0.003 (*p* < 0.0001**)	CP = 0.13 ± 0.011 NC = 0.07 ± 0.013 (*p < 0.0001**)	CP = 0.09 ± 0.006 NC = 0.11 ± 0.007 (0.067)	CP = 0.05 ± 0.004 NC = 0.07 ± 0.005 (0.012*)	CP = 0.07 ± 0.004 NC = 0.09 ± 0.005 (0.081)	CP = 0.05 ± 0.007 NC = 0.01 ± 0.009 (0.002*)	CP = 0.03 ± 0.003 NC = 0.02 ± 0.004 (0.089)

CP cerebral palsy, NC healthy children, Power Watt kg^−1^/m s^−1^, Work Joule kg^−1^/m s^−1^

## Discussion

### Loading Response

The positive power on initial contact with the ground normally means the knee is helping to lift the body and move forward while negative power means the knee is working passively. Both knees in children with CP were absorbing less energy (negative work) from the neighbouring joints than the healthy group while the children were contacting the ground. Minimum negative power in the CP group was roughly half of that in the healthy group during the loading response phase, indicating that the knee absorbed energy less in the CP than in the healthy group.

Approximately 50% more energy was generated in muscular contractions of the hip joint and more energy was transferred to the surrounding joints during loading response in the CP group (0.03 Joule kg^−1^/m s^−1^) compared to the healthy group (0.02 Joule kg^−1^/m s^−1^). These parameters were not improved in the post-treatment group of children with CP.

### Pre midstance

The knee joint normally attains a neutral position which is most stable during the pre midstance phase. Kinetic changes become zero or neutral. However, the minimum power in the CP group (− 0.57 Watt kg^−1^/m s^−1^) was approximately 67% of that seen in the healthy group (− 0.85 Watt kg^−1^/m s^−1^).

The hip normally assumes a neutral position in this phase. A higher positive value means that the hip muscles in children with CP were involved 4 times more (positive work) in stabilizing the joint than in the healthy group. A negative work implies that the hip was sometimes absorbing energy during walking and acting passively. A less negative value (one fourth) in the CP hip may be due to stiffness. Muscles around the hip joint generated more energy to maintain stability than absorbing energy from adjacent body segments during walking in the CP group. The minimum power was nearly three times greater in the healthy group than in the CP group. These parameters were not improved in the post-treatment group of children with CP.

### Post Midstance

Normally, ankle joint positive power reaches maximum to push the body forward. However, in this study, the ankle joint was absorbing approximately 50% more energy during this sub-phase, which was more apparent in the CP children (− 0.63 Watt kg^−1^/ms^−1^) than in the healthy children (− 0.35 Watt kg^−1^/ms^−1^). Ankle joint power sometimes became negative making it passive or losing control of movement. This appeared much more likely to happen in the CP children group compared to the healthy group.

Knee joint extension moment normally reduces by a small amount. Approximately 60% more negative work found in the CP group means the movement was passive or there was residual stiffness present.

Hip joint normal extension moment reaches maximum. In this study, hip muscles were generating nearly six times more work in the CP group to create effective extension than in the healthy children group. The hip was absorbing energy during extension approximately 30% less negative value in children with CP than in the healthy children and may be due to stiffness. The hip joint extensors in the CP group were generating nearly 5 times more power (Maximum) than the healthy group. These parameters did not improve in post-treatment group of CP children.

### Terminal Stance

Ankle power is positive to push the body forward in a normal gait cycle. The healthy ankle produced approximately 50% more energy than the CP ankle. Maximum power was approximately 60% more in the healthy group (2.77 Watt kg^−1^/ms^−1^) than in the CP group (1.72 Watt kg^−1^/ms^−1^). This was due to weakness in the muscles of the CP group.

The knee joint straightens for toe-off the ground in a normal gait cycle. The healthy group was 68% more (negative work) capable of absorbing energy than the CP group. Absolute work done by the healthy group was approximately 60% more than the CP group.

Hip joint positive power reaches a maximum in the terminal stance phase to push the body forward in a normal gait cycle. The healthy hip generated approximately 70% more energy during maximum extension than that seen in the CP group whose absolute work was approximately 70% of that of the healthy group. CP children generated maximum power by 67% of that of the healthy group in terminal stance.

### Due to space limitations, the analysis of the swing phase was not investigated

A previous study by Moreira et al. (2017) determined that Peak Hip Power (PHP) and PHP occurred in less than 68% of gait cycle time as sensitive indicators for decision making in Rectus Femoris Transfer surgery [20]. Another study by Ishihara and Higuchi (2014) formulated peak ankle power by peak hip power A2/H3 ratio based on graphs [14]. However, the present study noted the specific sub-phase in which the Peak Hip Power and Peak Ankle Power was appearing and had given quantitative values for easy comparison between pre and post-treatment kinetic parameters. Maximum hip power (Mean = 0.97 Watt/Kg/ms^−1^) and knee power (1.16 Watt/Kg/ms^−1^) in the CP group were both recorded during the pre-midstance phase. Maximum ankle power (1.72 Watt/Kg/ms^−1^) was recorded during the terminal stance phase of the gait cycle in the CP group. Dohin and Salem (2015) highlighted that the power was generated in the hip and knee in normal gait, so examination of hip and knee power before and after SEMLS surgery could aid in decision making in undertaking this surgery [15]. Their finding was that multi-level surgery had failed in restoring ankle power. The hip joint power acted as the main propulsive force after surgery. The findings of the present study concerning hip and ankle power generation after operative procedures were consistent with the previous study. There was significantly less power generation in the ankle joint. Several kinetic indexes and ratios for post-surgical evaluation of CP children could be formulated with regard to the study by Cimolin et al. (2018) [21].

### Limitation of Study

The sample size could have been increased to enhance the results if available. In the database, a lot of CP patients cannot independently walk without walking aids, which made it difficult in collecting more samples. We did recognise that the CP group had different situations and were mixed in the study. This seems to be a shortcoming, but this mixing group gave us a general trend for the population with CP, which is what we did intended to investigate. If the CP group had been further divided into several sub-groups depending on treatment and segment, the sample size would not have been available for any group study. It is recommended that in the future the gait data from multi-centres are available to investigate a larger CP group with identical symptoms and treatments.

### Clinical Relevance

The results from this study provide a database to highlight how different the CP and healthy control groups are in terms of work and power, which has been neglected in current clinical gait analysis. The database could be used in the assessment of gait for children with CP, e.g., whether the treatments make the gait toward the direction of healthy children; or whether rehabilitation improves the gait compared with the healthy children. Therefore, the database would be useful to clinical consultants and physiotherapists.

## Conclusions

Firstly, in the present study, power (Watt /kg^−1^ ms^−1^) values from the database were used for the calculation of work done (Joule /kg^−1^ ms^−1^) in different sub-phases of the gait cycle. Work done is equivalent to energy exchange (generation and absorption) by the joints in motion. CP children used energy differently. Now, it can be explained that walking speed in CP was lower due to different distributions of work done or energy exchanged in different sub-phases of the gait cycle. Secondly, it was found that the ankle joint maximum power generation was significantly lower in the CP group in terminal-stance than that in the healthy group. Knee joint negative work in the CP group was lower than the healthy children group during terminal stance, due to the weakness of absorbing energy function. Hip joint positive work was greater in the CP group than in the healthy group during loading response, pre-mid, post-mid phases rather than in the terminal stance phase. Therefore, the present study proves that analysis of power distributions in the lower limb joints can be used as a quantitative tool to assess if gait is similar to the healthy so that a treatment or rehabilitation programme can be arranged.
